# Nutrition in the Intensive Care Unit—A Narrative Review

**DOI:** 10.3390/nu13082851

**Published:** 2021-08-19

**Authors:** Aileen Hill, Gunnar Elke, Arved Weimann

**Affiliations:** 1 Department of Intensive Care and Anesthesiology, University Hospital RWTH Aachen University, D-52074 Aachen, Germany; 2 Department of Anesthesiology and Intensive Care Medicine, University Medical Center Schleswig-Holstein, Campus Kiel, D-24105 Kiel, Germany; gunnar.elke@uksh.de; 3 Department of General, Visceral and Oncological Surgery, Surgical Intensive Care Unit, Klinikum St. Georg, D-04129 Leipzig, Germany

**Keywords:** medical nutrition therapy, critical care, enteral nutrition, parenteral nutrition, micronutrients, energy, protein, review

## Abstract

Background: While consent exists, that nutritional status has prognostic impact in the critically ill, the optimal feeding strategy has been a matter of debate. Methods: Narrative review of the recent evidence and international guideline recommendations focusing on basic principles of nutrition in the ICU and the treatment of specific patient groups. Covered topics are: the importance and diagnosis of malnutrition in the ICU, the optimal timing and route of nutrition, energy and protein requirements, the supplementation of specific nutrients, as well as monitoring and complications of a Medical Nutrition Therapy (MNT). Furthermore, this review summarizes the available evidence to optimize the MNT of patients grouped by primarily affected organ system. Results: Due to the considerable heterogeneity of the critically ill, MNT should be carefully adapted to the individual patient with special focus on phase of critical illness, metabolic tolerance, leading symptoms, and comorbidities. Conclusion: MNT in the ICU is complex and requiring an interdisciplinary approach and frequent reevaluation. The impact of personalized and disease-specific MNT on patient-centered clinical outcomes remains to be elucidated.

## 1. Introduction

Medical nutrition therapy (MNT) is an essential part of the care for critically ill patients, but the optimal feeding strategy for patients in the intensive care unit (ICU) is still debated and often remains a challenge for the ICU team in clinical practice. Recommendations for MNT in critically ill patients vary between guidelines of the DGEM (German Society for Nutritional Medicine) [1], the ESPEN (European Society of Enteral and Parenteral Nutrition) [2], the A.S.P.E.N (American Society of Enteral and Parenteral Nutrition) [3], and other societies [4,5], and their implementation into clinical practice may be considered a challenge [6]. This article summarizes current recommendations and discusses available evidence. The important questions “why”, “who”, “when”, “how”, and “with what” are answered to provide a pragmatic oversight. The optimal MNT for special patient groups in the ICU is presented grouped by organ systems.

This narrative review is a summary of current guideline recommendations and more recent evidence afterwards. For many aspects of the broad topic “nutrition in the ICU”, we can only give an overview in this manuscript and refer to other references for greater detail. Pragmatic recommendations are given as a summary at the end of each chapter and represent the author’s opinion in the attempt to help the clinician focus on the most important aspects of each section.

## 2. “Why?”—Nutritional Status as a Prognostic Factor

MNT in the ICU aims at avoiding malnutrition in primarily well-nourished patients and at preventing further deterioration of previously malnourished patients.

Malnutrition is a significant prognostic risk factor for critically ill patients, influencing major outcomes such as mortality, length of stay (LOS), duration of mechanical ventilation, and infection rates [7]. In a current meta-analysis of 20 studies with 1168 patients, the prevalence of malnutrition in ICU patients was 38–78% [7], highlighting the need of optimal and individually tailored MNT in the ICU. In the same vein, the large percentage of malnourished patients makes nutritional risk assessment upon ICU-admission followed by an appropriate MNT mandatory. Special attention and care should be attributed to those patients who will be anticipated to stay for longer than a week in the ICU [8,9].

Feeding protocols should be used and have proven beneficial in the nutrition of ICU patients [10,11,12]. A clearly defined feeding protocol has shown to decrease the rate of patients who cannot be enterally fed at all and will increase the delivery of calories [13]. Protocols should be locally tailored according to expertise, local barriers, facilities, and patient subpopulation in the ICU [14]

**Pragmatic recommendations:**

- **Nutrition is a prognostically relevant part of ICU treatment and requires special care**
- **Standard operating protocols improve the local MNT**

## 3. “Who?”—Assessment of Nutritional Status

Pragmatically, when admitted to the ICU, according to Sundstrom et al., three patient groups may be metabolically differentiated (although the discrimination between them is still debated) [15]:

1. Patients in whom multi-organ insufficiency or -failure cannot be prevented or broken through by MNT
2. Patients who recover quickly and do not require MNT
3. Patients in whom the disease course may be positively influenced by an individualized MNT

The latter may be either patients with pre-existing malnutrition or patients with anticipated complicative courses and prolonged ICU stays. These patients benefit from an individually adjusted MNT, but their distinction from the other patient groups is not trivial. While it may be easy to identify an undernourished patient with low body weight and body mass index (BMI) during clinical routine, in case of normal or even elevated BMI, the detection of malnutrition is less likely [3]. In 2019, the Global Leadership Initiative on Malnutrition (GLIM) developed a new definition of malnutrition based on phenotypic and etiologic criteria [16].

There are no validated and recommended tools to estimate the nutritional status of a critically ill patient. Therefore, in case of (semi-) elective admissions, a screening for malnutrition in standard care before major surgery may be practical and is recommended [12]. Several tools to estimate nutrition risk are the Nutrition Risk Score (NRS 2002), the NUTRIC (Nutrition Risk in the Critically Ill) Score, the Subjective Global Assessment (SGA), or the Malnutrition Universal Screening Tool (MUST). In many of these tools, some of the following factors are included [16,17]:

- Medical history: age, comorbidities, loss of physical function
- Nutrition history: weight loss, reduced food intake, loss of appetite
- Physical examination: BMI, edema, body composition
- Severity of disease: critically ill patients are severely ill by definition

In many critically ill patients, the aforementioned parameters—such as nutrition history and exact BMI—are difficult to obtain. Weighing critically ill patients should be mandatory but feasibility may be limited in the ICU setting. In addition, changes of volume status impede weight measurements and clinical examinations, such as anthropometry [2]. The ESPEN guideline defines every patient who is in the ICU for more than 48 h to be at nutritional risk [2]. The DGEM guideline recommends a combination of low BMI, unintended weight loss and lack of oral food intake, or the SGA for critically ill patients [1].

Regarding technical tools, computed tomography (CT) scan analysis, musculoskeletal ultrasound, and bioelectrical impedance analysis (BIA) may be available for the assessment and monitoring of nutritional status in the ICU, but are not broadly implemented into clinical routine until now [18]. 

**Pragmatic recommendations:**

- **There is no single “golden bullet” to diagnose malnutrition, but many helpful tools and criteria**
- **All ICU patients should be regularly screened for risk of malnutrition**

## 4. “How?”—The Route of Nutrition

### 4.1. Enteral Nutrition (EN)

For a long time, the issue of “enteral or parenteral” has been controversially and quite emotionally discussed [19,20]. Two large multicenter randomized controlled trials (RCTs) did not show any significant difference in mortality, while EN was associated with a higher risk for gastrointestinal (GI) complications [21,22]. An RCT by Harvey at al. including 2400 patients found no difference in 30-day mortality or infectious complications in ICU patients receiving either EN or PN, while patients receiving PN had significantly less vomiting and hypoglycaemia [21]. A more recent RCT led by Reignier et al. (NUTRIREA-2) recruiting 2410 patients also observed no difference in 28-day mortality and infection rates, but significant more frequent GI complications (vomiting, bowel ischemia, and pseudo-obstruction) [22]

The current international nutrition guidelines uniformly recommend the preferential use of EN in the critically ill patient who is unable to maintain sufficient oral intake [2,3,4,23]. The physiological advantages, the paradigm of “use the gut or lose it”, adverse effects of PN in earlier decades and increased cost effectiveness led to uniform preference of EN. However, EN alone is often insufficient to achieve energy and protein targets particularly in the acute phase of critical illness due to frequent interruptions for procedures and/or GI intolerance [24], which a recent meta-analysis of EN+PN versus EN alone demonstrated [25].

Although clear benefits are lacking regarding the optimal nutritional route, worldwide consensus among experts exists about a cautious individualized approach with ‘trophic feeding’ in high-risk patients without absolute contraindication followed by a ramp-up strategy until the target is reached [20]. While severe critical illness is frequently associated with considerable GI dysfunction, even severe sepsis or septic shock have not been considered clear contraindications in the guidelines [1,2,3,4]. EN can be administered via nasogastric or nasojejunal tube. If the need for EN will potentially exceed four weeks, placement of percutaneus endoscopic gastrostomy/jejunostomy is recommended [23].

Contraindications for EN according to the European Society of Intensive Care Medicine (ESICM) Clinical Practice Guidelines [4] particularly include hemodynamic instability (escalation of or high vasopressor medication and increased lactate) and GI intolerance from minor to major symptoms, e.g., gastric residual volume (GRV) > 500 mL/6 h or acute gastrointestinal injury grade > 2.

### 4.2. Combination of EN with Parenteral Nutrion (PN)

The progression of EN up to calorie/protein target is often prevented by feeding intolerance or common interruptions of EN [26,27,28]. Thus, particularly in the patient’s first ICU week, EN alone may lead to macronutrient deficiency [29,30,31]. To avoid large cumulative energy and protein deficits, EN and PN may be combined, either early during the patient’s ICU course (combined EN+PN), or after several days once EN is proven to be insufficient or unfeasible (supplementary PN [SPN]) [19]. 

The debate between early or late PN in addition to EN remains controversial [32,33,34]. There are strong arguments to start PN latest by day 4 particularly in malnourished patients and those with special risks [34], respecting potential risk of refeeding-syndrome.

A recent meta-analysis by Hill et al. including 12 RCTs with 5543 patients found that treatment with combined EN and PN led to increased delivery of macronutrients in severely ill ICU patients [25]. No statistically significant effect of a combination of EN with PN vs. EN alone on any of the analyzed endpoints were observed: mortality (Risk Ratio [RR] 1.0, 95% Confidence Interval [CI], 0.79 to 1.28 *p* = 0.99), hospital LOS (Mean Difference [MD]-1.44, CI −5.59 to 2.71, *p* = 0.50), ICU LOS and ventilation days. Trends toward improved physical outcomes were observed in two of four trials. There was a tendency for reduced mortality in nutritionally at-risk patients in some subgroup analyses, but data were too sparse to draw further conclusions. 

The ESPEN guideline recommends as good practice point: “PN should not be started until all strategies to maximize EN tolerance have been attempted” and “In patients who do not tolerate full dose EN during the first week in the ICU, the safety and benefits of initiating should be weighed on a case-by-case basis” [2].

**Pragmatic recommendations:**

- **EN may be preferred in almost all ICU patients**
- **Macronutrient targets may not be reached with EN alone in the acute phase**
- **The addition of PN or the use of total PN (in the acute phase) needs to be considered on a case-by-case basis**

## 5. “How Much?”—Energy and Protein Requirements

It remains unclear what the optimal protein energy targets should be and exactly when they should be reached [35,36,37]. Greater protein and energy intake may be associated with improved mortality in patients at nutritional risk as stated in a recent meta-analysis, but evidence remains controversial [25]. The number of macronutrients for critically ill patients needs to be carefully adapted to the individual patient [1]. Aspects requiring careful consideration and regular re-evaluation are the phase of critical illness (acute vs. post-acute), GI and metabolic tolerance to exogenous substrates, the primary disease, possible comorbidities, macro- and micronutrient deficiencies, and the intraindividual disease trajectory of the patient [1,2,3]. Tailoring the MNT to the individual patient´s metabolic tolerance is described below.

### 5.1. Energy

The optimal amount of energy is not yet agreed upon, as the evidence remains conflicting. What the targets should be and when they should be reached is still unclear, especially in the acute phase of critical illness, as targeting only caloric adequacy did not show statistically significant improvements in many studies [19]. 

The guidelines uniformly recommend using indirect calorimetry to determine energy requirements [1,2,3]. Recent advances in this technique and development of modern devices may improve feasibility and usefulness in clinical practice [38]. Two meta-analyses from 2021 concluded that patients treated with calorimetry-guided isocaloric nutrition had significantly lower short term mortality rates [39,40]. Aiming at personalized nutrition, these results may further stimulate the use of indirect calorimetry in ICUs worldwide.

Otherwise, weight adapted formulas may be used but may only be considered an alternative. The general recommended calculated energy targets vary between guidelines and are 24–30 kcal/kg/d, see Table A1, Appendix A [1,2,3].

### 5.2. Protein

The guidelines currently recommend a protein target of 1.0–2 g/kg/d, see Table A1, Appendix A [1,2,3], but the influence of protein on the outcome of critically ill patients has also been discussed controversially [41,42]. Increased protein intake, was associated with improved long-term physical recovery and lower mortality in observational trials [43,44,45,46] and did not influence duration of renal dysfunction [47]. However, a systematic review and meta-analysis of 14 RCTs did not show any impact of different amounts of protein delivery on outcomes mortality, mechanical ventilation, infections, and length of stay [48]. It must be noted that the protein delivery of the included trials was below the guideline recommendations (mean: 67.15 g vs. 42.95 g/d). Suppressed autophagy has been discussed to explain the discrepancy between observational studies and the meta-analysis [49]. 

The most recent evidence from RCTs regarding protein showed no significant differences in clinical outcomes. Targeting full energy and protein delivery a RCT (FEED) including 60 patients had shown significantly attenuated muscle loss and a lower number of malnourished patients [50]. In an RCT from 2021, patients receiving higher protein delivery (1.5 ± 0.5 g vs. 1.0 ± 0.5 g/kg_BW_/d) did not show different clinical outcomes or changes in quadriceps muscle layer thickness [51]. For comparison of protein delivery according to the international guidelines vs. usual care, a recent multicenter RCT including 120 patients investigated the feasibility of a high-protein enteral nutrition formula (100 g/L vs. 63 g/L). The two groups were comparable in energy delivery as well. Protein delivery in the intervention group was higher (1.52 vs. 0.99 g/kg ideal body weight per day). No differences in the clinical outcomes including 90-day mortality were observed [52].

Even in nutrition trials targeting the adequate provision of protein, EN failed to provide more than 1.5 g/kg/d in all the mentioned trials [53]. A very recent meta-analysis concluded that a higher versus a lower protein delivery (1.31 ± 0.48 vs. 0.90 ± 0.30 g/kg) did not significantly influence overall mortality (RR 0.91, 95% CI -0.75 to 1.10, *p* = 0.34) or other outcomes [54]. Large trials, such as the EFFORT Trial (NCT03160547) are currently ongoing to evaluate the influence of high and low protein dosages in critically ill [41].

### 5.3. Non-Nutritional Calories

Calculation of total calorie intake should include intake of non-nutritional calories. Depending on the dose, propofol used for sedation patients may represent a significant portion of the total calorie intake. 1% and 2% propofol contains 0.1 g fat/mL; at a propofol infusion rate of 20 mL/h, fat intake would be 48 g fat/d; thus, about 450 kcal/d would be additionally provided. In a retrospective analysis of 687 critically ill patients, sedation with propofol resulted in an additional calorie intake of 146 ± 117 kcal/d, corresponding to 17% of total calorie intake [55].

Trisodium citrate is commonly used for regional anti-coagulation during renal replacement therapy (RRT). The number of effective calories provided by citrate depends on the citrate concentration /infusion rate, the blood flow rate, the filtration fraction of the ultrafiltrate per unit time and type of filter. For example, a trisodium citrate solution may contain 0.59 kcal/mmol. An infusion rate of 11–20 mmol/h according to 3 kcal/g would result in a caloric intake of 150–280 kcal/d.

A retrospective study of 146 critically ill patients showed that the median propofol and citrate contribution to total calorie intake was 6–18% during the first seven days after ICU admission. In individual cases, however, this portion may increase up to one-third of total calorie intake [56].

**Pragmatic recommendations:**

- **Optimal macronutrient targets are controversially debated**
- **Prefer indirect calorimetry to measure energy expenditure**
- **Weight-adapted formulas may be used alternatively:**
  ◦ **Energy: 24–30 kcal/kg/d**
  ◦ **Protein: 1.0–2 g/kg/d**
- **Do not neglect non-nutritional calories**

## 6. “When?”—The Timing of Nutrition

### 6.1. Early EN (EEN)

The early initiation of EEN within 24–48 h is uniformly recommended by the guidelines in the critically ill patient who is unable to maintain sufficient oral intake [2,3,4,23]. Bearing in mind the traditional concept that the gut may be the “motor” for multi-organ dysfunction, EN should be started at a low feeding rate (e.g. 5–10 mL) and increased carefully and individually adapted to hemodynamic stability and tolerance [12].

Regarding the effect of EEN, a meta-analysis by Tian et al. [57] (8 RCTs, 1895 patients) calculated a significantly decreased lower rate of mortality (RR 0.68; 95% CI, 0.51 to 0.92; *p* = 0.01) and GI intolerance (RR 0.65; 95% CI 0.43 to 0.99; *p* = 0.05) in a subgroup of patients with low energy intake (33.3–66.6% of energy target). However, GI intolerance was only reported in three studies, including 452 patients. 

Comparing EEN and PN four recent meta-analyses [35,57,58,59] including up to 25 studies with 3816 patients provided these results:

- In general, EEN has no significant impact on mortality, but this may be reconsidered in high-risk subgroups.
- EEN significantly decreases the risk for infectious complications.

### 6.2. How Early Is Too Early to Add PN?

Regarding the exact timing of initiating PN, the recommendations are contradictory. The A.S.P.E.N guideline recommends that PN should be withheld in patients at low nutrition risk during the first 7 days following ICU admission [3]. The ESPEN guideline advises implementing PN within 3–7 days in these patients if EN is contraindicated [2]. The DGEM does not address a particular recommendation regarding the timing of starting PN but recommends to already use PN in the acute phase if calorie-protein targets cannot be reached by EN alone [1]. 

However, in severely malnourished patients or patients at high nutrition risk, ESPEN and A.S.P.E.N guidelines state that early and progressive PN should be provided to patients with contraindications for EN [2,3]. The DGEM guideline states that PN may be the better route for malnourished patients, because they frequently experience GI dysfunctions [1]. 

The above-mentioned meta-analysis from Hill et al. did not detect any clinical differences between an early combined EN+PN or the addition of SPN several days after ICU admission regarding mortality, ICU and hospital LOS or mechanical ventilation [25].

Therefore, it is obvious that the indication for PN in the critically ill has become more critical and individualized. The ESPEN guideline states “In patients who do not tolerate full dose EN during the first week in the ICU, the safety and benefits of initiating should be weighed on a case-by-case basis” and adds a practical point: “PN should not be started until all strategies to maximize EN tolerance have been attempted” [2].

**Pragmatic recommendations:**

- **Initiate EN within 24–48 h in patients without contraindications**
- **Optimize EN delivery**
- **The addition of PN needs individual adaptation**

## 7. “What?”—Formula Considerations

In most critically ill patients, standard polymeric formulas for EN should be used according to the guidelines [3,12]. For PN, all-in-one bags should be preferred [12].

### 7.1. Energy-Dense versus Standard Formula

Due to impaired GI tolerance in the critically ill, energy-dense EN may theoretically improve nutrient delivery. Two multicenter RCTs concluded that energy dense EN (1.5 kcal/mL) increased macronutrient delivery, while adverse effects were not increased [60,61]. However, in the latter trial [61], the need for insulin was higher in the 1.5 kcal group and the 90-day survival was not significantly different. In a recent RCT, scintigraphic measurement of gastric retention at 120 min was greater in the group with the energy-dense formula, intestinal energy delivery and glucose absorption were not improved [52].

### 7.2. Special Enteral Diets: Synbiotics

Synbiotics refer to the combination of both probiotics and prebiotics, containing Lactobacillus organisms alongside fiber. Synbiotics reveal trophic effects in the colon, focusing on the preservation of the microbiome, promoting mucosal regeneration in balance with the microenvironment. Their administration is a challenging concept with a potential impact on diarrhea and infectious complications [62,63].

Results from current evidence are equivocal. Including nine studies, a meta-analysis from Batra et al., showed a reduction of the incidence of pneumonia (RR: 0.70, CI 0.56 to 0.88; *p =* 0.002), the duration of mechanical ventilation (MD −3.75, CI −6.93 to −0.58; *p =* 0.02), ICU LOS (MD −4.20, CI −6.73 to −1.66; *p* = 0.001), and in-hospital mortality (OR 0.73, CI 0.54 to 0.98; *p* = 0.04) [64]. A more recent meta-analysis emphasized the considerable heterogeneity of the studies. While no benefits for clinical outcome parameters were found, reduced prevalence or duration of diarrhea episodes were observed [65]. A very recent RCT including 218 patients did not confirm a significant difference in infectious complications [66].

The A.S.P.E.N. guidelines suggest that a commercial mixed fiber formula should not be used routinely in the adult critically ill patient prophylactically to promote bowel regularity or prevent diarrhea [3]. The German DGEM guidelines recommend probiotics for patients with severe trauma and those undergoing liver transplantation [1].

**Pragmatic recommendations:**

- **Standard formulas should be chosen for most ICU patients**
- **Consider energy dense formulas in patients with fluid restrictions and in malnourished patients**
- **Intensified blood sugar monitoring may be beneficial in patients with energy dense formulas**

## 8. Supplementation with Specific Nutrients

The supplementation of EN or PN with immune-enhancing and anti-inflammatory nutrients is a challenging and controversial issue. 

### 8.1. Arginine, Glutamine, and Omega-3 Fatty Acids

The guidelines currently do not recommend the use of arginine, glutamine, and omega-3 fatty acids in the general critically ill patient population. 

Regarding arginine, in theory, the availability is reduced in sepsis, but as arginine supplementation could induce the formation of nitric oxide and increase hypotension in patients with septic shock, there is limited data [67,68].

In critical illness, the glutamine concentration in plasma may be reduced and considered an expression of the severity of the disease and infection [69]. Supplementation of glutamine may possibly lead to a reduction in bacterial translocation from the gut, improved immune cell function, decreased proinflammatory cytokine production, and increased antioxidant capacity. The clinical impact of these findings, however, has not yet been clearly established and in most meta-analyses, no statistical significance was reached. Because a high dosage in patients with (multi-)organ dysfunction may be detrimental [70,71], in unstable and complex ICU patients, particularly in those suffering from liver and renal failure, parenteral glutamine shall not be administered [2]. An “umbrella” overview of 22 meta-analyses and a current meta-analysis of 15 randomized studies have shown again benefits for glutamine supplementation in ICU patients with regard to the rate of infectious complications and the hospital LOS [72,73,74]. Reference was also made to the considerable and statistically significant heterogeneity of the studies and meta-analyses [72]. The new ESPEN practical guideline “clinical nutrition in surgery” states parenteral glutamine may be considered in patients requiring exclusive PN [12].

Fish oil or ω-3 fatty acids may have a high anti-inflammatory potential due to the shift in inflammatory mediator synthesis but may intensify an already existing immunosuppression. A recent meta-analysis of 49 RCTs, risk of infection was 40% lower with ω-3 fatty-acid enriched PN than standard PN (RR 0.60, 95% CI 0.49 to 0.72; *p* < 0.00001). Patients given ω-3 fatty-acid enriched PN had reduced ICU LOS (*p* = 0.01) and reduced length of hospital LOS (*p* < 0.00001). Risk of sepsis (9 RCTs) was reduced by 56% in those given ω-3 fatty-acid enriched PN (RR 0.44, 95% CI 0.28–0.70; *p* = 0.0004) [75]. Focusing on enrichment with omega-3 fatty acids in EN and supplemental PN in a recent RCT with 100 mechanically ventilated ICU patients, neither improvement in lung function, nor decrease of complications were observed. However, the supplemented group could be weaned earlier from catecholamine treatment and PN [76]. In patients requiring PN, the new ESPEN practical guideline “clinical nutrition in surgery” states postoperative PN including ω-3 fatty acids should be considered [12]. 

### 8.2. Selenium

Selenium is a known antioxidant and decreases in septic patients and after major surgery. Current evidence for the use of intravenous selenium has been inconclusive. An RCT (SISPCT) administering high-dose selenium to 1089 patients with severe sepsis or septic shock, the 28-day mortality rate in the selenium group was not significantly affected (*p* = 0.30) [77]. A recent meta-analysis focusing on eight RCTs with low risk of bias did not find effects of antioxidant micronutrient supplementation on the reduction of mortality [78]. Currently, an international multicenter RCT of selenium in cardiac surgery (SUSTAIN-CSX, NCT02002247) is undergoing statistical evaluation [79].

Currently the guidelines do not recommend pharmacological supplementation with selenium [1,2].

### 8.3. Vitamin D

Vitamin D exerts pleiotropic effects with actions far beyond its classic role in mineral homeostasis. Tissue actions require two enzymatic conversions to 25-hydroxyvitamin D and 1,25-dihydroxy (OH) vitamin D after which vitamin D has been shown to modulate the immune response amongst others. Vitamin D deficiency is highly prevalent across all age groups and countries [80]. Although vitamin D deficiency was further determined to be associated with greater illness severity (also in patients with COVID 19), a causal relationship between vitamin D deficiency and multiple organ dysfunction has not been established [81,82,83,84]. Thus, the efficacy of vitamin D as a therapeutic in critically ill patients remains controversial [85].

While in a previous phase 2 trial (underpowered for mortality as endpoint [VITdAL-ICU], involving 475 patients), vitamin D supplementation administered to vitamin D–deficient, critically ill patients was associated with lower observed mortality than placebo at 28 days (21.9% vs. 28.6%, *p* = 0.14) and at 6 months (35.0% vs. 42.9%, *p* = 0.09) [86]. The subsequent randomized, double-blind, placebo-controlled, phase 3 (VIOLET) trial including 1360 patients (1078 vitamin D deficient at baseline defined as 25-hydroxyvitamin D level, <20 ng/mL) receiving a single enteral dose of 540,000 IU of vitamin D or matched placebo did not reveal clinically important differences between the groups with respect to secondary clinical, physiological, or safety end points. The severity of vitamin D deficiency at baseline did not affect the association between the treatment assignment and mortality.

In a recent sub-study of the VIOLET trial, long-term cognitive outcomes were measured. The adjusted median score at follow-up (median 443 days) was not significantly different (adjusted OR, 0.83; 95% CI, 0.50 to 1.38) [87].

Another randomized, placebo-controlled, double-blind, multicenter, international trial, is ongoing with planned recruitment of 2400 adult patients with severe vitamin D deficiency (25-OH Vit D ≤ 12 ng/mL) receiving a enteral loading dose of 540,000 IU cholecalciferol within 72 h after ICU admission, followed by 4000 IU daily for 90 days or placebo [88].

So far, there is no clear evidence for pharmacological vitamin D supplementation in patients with established deficiency. 

Given the controversial evidence, different recommendations can be found among the guidelines (1, 2) (see Table 1).

### 8.4. Vitamin C

Vitamin C is a pleiotropic nutrient and powerful antioxidant. While suboptimal vitamin C status is common among critically ill patients, vitamin C is currently not recommended as pharmacotherapy for these patients. Nevertheless, a very recent meta-analysis from Patel et al. (acceped for publication, registered at PROSPERO: CRD42021244074) could demonstrate with a trend towards reduction in overall mortality (RR 0.88, 95% CI 0.75 to 1.02, *p* = 0.09), which became significant when comparing high dose vitamin C supplementation (≥10,000 mg/d) with placebo (RR = 0.70, 95%, CI 0.52 to 0.96, *p* = 0.03). In the CITRIS-ALI Trial from Fowler et al., in 2019, vitamin C–infused patients exhibited a significant reduction in 28-day all-cause mortality (χ^2^ = 4.84; *p* = 0.03) [89].

Regarding the “Marik cocktail”, a treatment with intravenous hydrocortisone, ascorbic acid (vitamin C) and thiamine (HAT) [90], a current meta-analysis from 2021 in septic patients found no significant difference between both groups in long term mortality, ICU mortality, or incidence of acute kidney injury, hospital and ICU LOS, and ICU free days on day 28 between the intervention and control groups [91]. There was, however, a significant difference in the reduction of SOFA score on day 3 from baseline (MD −0.92; 95% CI −1.43 to −0.41; *p* < 0.05). 

In the before-mentioned meta-analysis (PROSPERO: CRD42021244074), vitamin C monotherapy was associated with a significant reduction in overall mortality (RR 0.66,95% CI 0.49 to 0.89, *p* = 0.006), while there was no effect on overall mortality in the trials administering vitamin C in combination with thiamine and hydrocortisone (RR 0.99, 95% CI 0.82 to 1.19, *p* = 0.91, test for subgroup differences was significant, *p* = 0.02).

**Pragmatic recommendations:**

- **The guidelines currently do not recommend pharmacotherapy with micronutrients in the general critically ill patient population.**
- **Proven micronutrient deficits need to be treated.**
- **Micronutrients must be supplemented whenever PN is administered to a patient.**
- **While arginine and glutamine have no indication in the critically ill with special regard to those with organ failure, the enteral and parenteral supplementation of fish oil remains a matter of debate.**

## 9. Monitoring and Complications

In addition to the clinical examination of the patient’s abdomen, blood chemistry includes serum glucose, triglycerides, lactate, and procalcitonin should be measured. Phosphate should be controlled to detect and treat a potential refeeding syndrome. To avoid measurement of nitrogen balance, urea excretion rate per 24 h will help to estimate the extent of catabolism. For the estimation of intestinal function, the potential impact of biomarkers as citrulline and fatty acid binding protein plasma level have to be validated in clinical studies.

### 9.1. Gastrointestinal Intolerance

There is a variety of definitions for feeding intolerance including a lot of uncertainty for the clinical management [92]. Many critically ill patients experience feeding intolerance, motility disorders, which include delayed passage with slow gastric emptying and constipation, and accelerated passage with impaired small intestinal nutrient absorption or nutrition-related diarrhea [92,93]. According to the ERAS Society’s (Enhanced Recovery After Surgery) various guidelines [94,95,96], causes of GI intolerance include opioid analgesia, sedation, edema, and insufficient stimulation. Treatment options are oral/EN, prokinetics, mobilization and physical therapy, prokinetics and optimization of sedation and volume status. Clinical observation of the patient’s abdomen, bowel motility, and gastric reflux is mandatory. 

While measurement of GRV is not standardized the optimal threshold is uncertain. Therefore, the impact of the measurement of GRV has been controversial. The monitoring of GRV is considered still relevant in surgical ICU patients and severely critically ill patients with a high risk for GI dysfunction. A GRV of more than 500 mL/6 h may be considered critical [93]. In two controlled studies non-monitoring of GRV was without significant effect on the risk of ventilator-associated pneumonia in adults receiving mechanical ventilation and EEN [97,98].

Additional possibilities to monitor GI tolerance include sonography and CT scans. If the latter shows bowel loops with intramural air accumulation, bowel ischemia must be considered. Intramucosal phi tonometry as a tool for the measurement of splanchnic perfusion has not been used frequently in clinical practice. 

A research agenda has been proposed by the working group GI failure of the European Society of Intensive Care Medicine (ESCIM) including the core set of monitoring and outcomes [99]. 

### 9.2. Metabolic Intolerance

Hyperglycemia may be due either to hyperalimentation in the (acute) phase of insulin resistance or underlying subclinical diabetes. Hyperglycemia is associated with increased mortality, which has been the rationale for intensified insulin therapy [100]. To avoid any life-threatening hypoglycemia, nowadays, glucose levels up to 180 mg/dL (10 mmol/L) are accepted [5,101]. Reduction of glucose calories should be considered before insulin is administered in nondiabetic patients with moderate dosages of 0–4 IU/h. An intensive insulin therapy is not recommended to avoid the potential consequences of hypoglycemia [5]. 

To circumvent hypertriglyceridemia, bolus application of lipids should be avoided. During continuous lipid infusion serum triglyceride levels should not exceed 400 mg/dL [101]. Reversible liver steatosis or hypertriglyceridemia-induced acute pancreatitis may develop if metabolic control cannot be achieved within several days.

In severely malnourished patients and patients after long starving periods, a refeeding syndrome may occur within the first few days after starting MNT. Supply of carbohydrates and fluid will stimulate insulin secretion and intracellular shift of glucose and electrolytes. The decrease of serum potassium, magnesium, and phosphate levels will lead to impaired neuromuscular transmission causing life-threatening arrhythmias, and convulsions. Calories and fluid administration should be increased slowly under electrolyte- and preferably ECG monitoring [102]. In high-risk patients, a preventive thiamine administration 200 mg once daily for 2 days is necessary. 

**Pragmatic recommendations:**

- **Clinical examination, bowel motility, and gastric reflux and GRV are helpful to assess gastrointestinal tolerance of EN.**
- **Optimization of analgosedation, fluid status, and GI-stimulation can improve the tolerance of EN**
- **Markers of metabolic tolerance include blood sugar, lipids, and electrolytes with special focus on potassium, magnesium, and phosphate**

## 10. Tailoring MNT to the Individual Patient’s Needs

**Figure 1:**
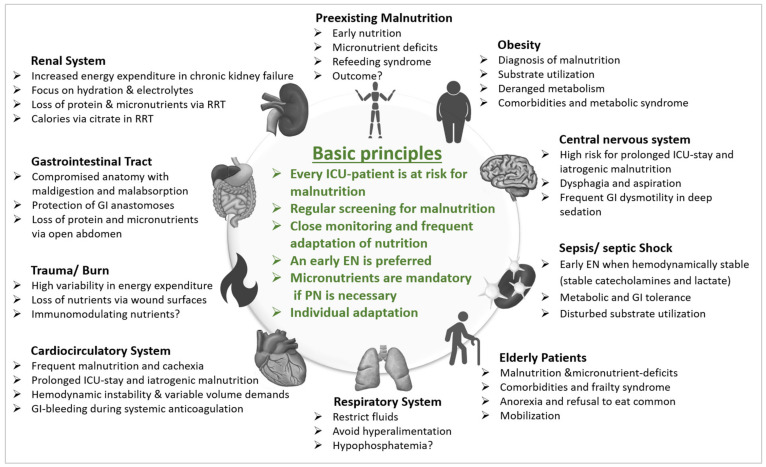
Overview important aspects of medical nutrition therapy in different patient groups. Abbreviations: EN: enteral nutrition, GI: gastrointestinal, PN: parenteral nutrition, RRT: renal replacement therapy.

The heterogeneity of the patients in the ICU complicates MNT and many different factors such as primary disease, comorbidities, phase of critical illness influence the patient’s individual requirements. The most important aspects of the different patient groups are summarized in Figure 1 to give a pragmatic overview. 

### 10.1. Adapting MNT to the Phase of Critical Illness

MNT must be adjusted to the metabolic tolerance which may considerably alter in the different phases of critical illness and resulting catabolic response [1,2]. While metabolic tolerance may be extremely limited by severe inflammation in the early acute phase bearing the high risk of overfeeding, it is different in the post-acute phase according to chronical inflammation or beginning resolution and recovery—the shift to anabolism includes the risk of underfeeding. Therefore, the guidelines recommend an individualized approach due to specific pathophysiologic and resulting metabolic changes, the guidelines recommend an individual adaptation of MNT to the different phases of critical illness [1,2].

Immediately after onset of the critical illness, the ‘acute’ phase begins that can be divided into an ‘early acute’ phase (about 1–3 days post-onset with the possibility of fatality due to the most severe illness entity) and a ‘late acute’ phase (approximately lasting for 2–4 days if the patient survives the early acute phase). The post-acute phase can be described as a ‘recovery’ phase (duration >7 days), which is usually spent in the primary care hospital. During this phase of recovery from catabolic critical illness, substrate tolerance has been normalized with a metabolic shift to anabolism. From a nutritional point of view, sufficient macronutrient supply in this period may be considered pivotal to the patient’s recovery and long-term outcome. The amount of administered calories are considered to be 1.2–1.5 fold the calculated energy requirement. Because these patients are weaned from ventilation and able to eat, it will be frequently—and falsely—assumed that they will manage to cover these requirements by the oral route. It has been recently shown that this may be achieved only by a combination of oral and enteral feeding [103]. It has been shown that after extubating, many patients will receive no more than 700 kcal/d [104]. Reasons may be early discontinuation of MNT in favor of oral nutrition (especially in case of discharge to standard care), limited oral intake due to post-critical weakness, fatigue, anorexia, and isolation. Documentation of the amount of oral intake is mandatory, supplementation with oral nutritional supplements (ONS), in some cases enteral or even SPN should be considered. 

After the recovery phase, the ‘rehabilitation’ phase (lasting several months) follows, in which, among others, the metabolic damage suffered initially is repaired slowly. Usually, patients will not go through this phase in the primary care hospital. Alternatively, the ‘post-acute’ phase may merge into a ‘chronic’ phase (of uncertain duration) characterized by persistent organ dysfunction and an uncertain prognosis. This particular course may be described as a “persistent inflammatory immunosuppressed catabolism syndrome” [105,106]. If there is a new disturbance of homeostasis, the process will start again with the acute phase.

**Pragmatic recommendations:**

- **Adapt MNT to the phase of illness**
- **In the early acute phase, a lower supply of macronutrients may be beneficial**
- **In the postacute phase, reaching nutritional targets may be of great importance for the patient’s long-term outcome**

### 10.2. Critically Ill Patients with Altered Nutritional Status

Malnutrition is a common phenomenon and includes under- as well as overnutrition. While undernourished patients with low weight and BMI can be easily recognized during clinical routine, malnutrition may be underestimated in patients with normal or elevated BMI [3]. An obese patient with weigh loss in the past months or with reduced intake of nutrition is at risk for malnutrition, which may not be diagnosed at first sight. Therefore, a combination of clinical evaluation and the use of a validated screening tool may be recommended [1,2,3,107]. If the patient is at nutritional risk, a detailed nutritional assessment should follow.

#### 10.2.1. Undernourished Patients

In patients with preexisting malnutrition, according to the DGEM guideline, the same energy and protein targets may be used, as in other patients, as there is still a lack of data concerning the therapeutic relevance of MNT in this patient cohort although the prognostic relevance of malnutrition is clear [1]. In these patients, an MNT should be commenced early to avoid large cumulative macronutrient debts. This may be achieved through ONS or though SPN. Should an EN not be feasible, an early hypocaloric PN should be administered (75% of caloric target, protein ≥ 1 g/kg/d) [1].

In severely malnourished patients, according to the DGEM guideline, a more aggressive MNT is not recommended to avoid GI and metabolic intolerance and potential complications such as acute hyperalimentation, refeeding syndrome, or increased rates of infection [1].

Contrasting this recommendation, the A.S.P.E.N. suggests a rapid progression of preferably EN with the aim to reach the target within 24–48 h under careful monitoring. Within 48–72 h, >80% of energy and protein goals should be achieved [3]. However, these recommendations of the A.S.P.E.N. rely on debatable evidence.

While the essential role of micronutrients for many biological processes in the human body is accentuated in current literature, reliable evidence remains sparse. Therefore, micronutrients in pharmacological dosages should only be administered after clinically raised assumptions or measured micronutrient deficiencies as stated by the DGEM [1].

Special attention should be paid to the occurrence of a refeeding syndrome in severely malnourished patients.

#### 10.2.2. Obese Patients

Obese patients do not have ‘reserves’, as often assumed, but in contrast they frequently suffer from disturbances in substrate utilization, leading this patient group to be predisposed to loss of muscle mass during an ICU stay. Increased attention should be paid to the monitoring of metabolism, markers of metabolic syndrome and possible comorbidities [3]. While there is only weak evidence, the goal of an MNT is avoidance of muscle catabolism, improvement in body composition, reduction of insulin resistance and hyperglycemia and reduction of infection rates. 

For obese patients with BMI ≥ 30 kg/m^2^, similar guideline recommendations were issued as for other ICU patients [1,2,3]. The best MNT for obese patients is still debated particularly because evidence remains weak from smaller and quite older observational studies, as are formulas to calculate energy demand in these patients, but indirect calorimetry should be used to measure EE [1,2]. Otherwise, obese patients should be nourished weight-adapted hypocalorically and high in protein (1.5 g protein (1.8 g amino acids)/kg_ideal BW_/d) [1,2,3,108]. For calculation of ideal BW, the so-called Peterson formula is recommended in a slightly different modification by the ESPEN and DGEM guideline (DGEM: ideal BW = 48.4 + 77.0 × (height-1.50 m) related to a BMI of 22 kg/m^2^; ESPEN: adjusted BW = (current BW-ideal BW) × 0.33 + ideal BW, ideal BW = 2.2 × BMI + 3.5 × BMI × (height-1.50 m) related to an “overweight” BMI of 25 kg/m^2^) [109]. In clinical routine, a hypocaloric high-protein nutrition may be achieved via enteral or parenteral protein supplements. Additionally, patients who experienced weight loss in the past months or who underwent bariatric surgery, should receive vitamin and trace element supplements with special focus on thiamine [1,3]. 

**Pragmatic recommendations:**

- **Diagnosis of malnutrition is not trivial and needs care**
- **In undernourished patients, an early MNT including ONS, EN, and PN may be considered with special attention to metabolic and GI feeding tolerance**
- **In overweight patients, an iso-proteinic/lower calorie approach might avoid hyperalimentation or muscle-catabolism**

### 10.3. Elderly Critically Ill Patients

In elderly patients, the most important goal is to optimize the functional capabilities to achieve the best possible quality of life and autonomy [110]. Elderly patients have higher incidences of comorbidities, malnutrition, sarcopenia, and cachexia, which may be regarded as the frailty syndrome [110]. Because the multidimensional syndrome “frailty” and other reasons for malnutrition, such as depression, anorexia, polypharmacy, low activity and catabolism of chronic diseases, and inflammation are so common in elderly patients and affects nutritional status, all elderly patients should be screened for malnutrition and be treated accordingly [111,112]. If the elderly critically ill patient is able to feed orally, sedative measures and dietary restrictions should be minimized to avoid a reduced food intake, which may negatively affect patient outcome [110]. In obese elderly patients, a weight-reducing diet should be circumvented, to avoid muscle catabolism [110]. Possibilities to optimize an oral nutrition include enriched nutrition, several small meals, ONS, monitoring of food intake and company during meals. If oral nutrition is insufficient, EN and or PN should be administered with the same indications as in other critically ill patients, while minding an early start of a MNT if indicated [110]. 

In the absence of methodologically sound clinical evidence, the DGEM chose not to give special recommendations for this patient group [1]. The ESPEN assumes that an adequate macronutrient supply may lower the incidence of frailty in elderly patients and recommends higher protein target (1.2–1.5 g/kg/d) for elderly malnourished patients [2]. In elderly critically ill patients a hypercaloric and high-protein supplementation (30 kcal/kg/d energy and ≥1 g/kg/d protein) is recommended by the ESPEN as well [110]. Micronutrients should be supplied as for healthy elderly patients, special micronutrient deficits shall be counterbalanced [110]. The A.S.P.E.N. does not provide special recommendations for elderly ICU patients.

In addition to an effective MNT, the focus should be on adequate hydration, potential refeeding syndrome [113], as well as on preservation of muscle mass physical activity to allow for an independent living after hospital discharge [110]. 

**Pragmatic recommendations:**

- **Malnutrition and dehydration are common among this group**
- **A critical (re-)evaluation of nutritional status is necessary**
- **Focus on the most physiologic route of nutrition, patient’s preferences, and function**
- **Consider a higher protein target to counteract anabolic resistance**

### 10.4. Critically Ill Patients in Shock

Physiologic advantages or an EN are the maintenance of the gut mucosa and the GI barrier, the modulation of an inflammatory reaction and the reduction of insulin resistance. In case of compromised hemodynamics, the concern for mesenterial ischemia due to the increased demands for the gut system is voiced in the guidelines [2,3]. Cautious EN in patients with the need for catecholamines or vasopressors may be considered [114], but there is still a risk for nonocclusive bowel disease and a lack of data from controlled randomized trials [115,116,117]. In the NUTRIREA-2 trial including 2410 patients, early EN did not reduce mortality or the risk of secondary infections but was associated with a greater risk of digestive complications compared with early isocaloric PN [22].

In the concept of “minimal trophic nutrition” it is recommended to start EN with a low flow rate (10–20 mL/h) [118]. In case of hemodynamic instability and the administration of catecholamines, limited enteral tolerance should be anticipated. Careful clinical examination of the abdomen must be performed and keeping in mind that the energy target cannot be achieved in such patients via the EN in most cases within the first ICU week. Complete stop of enteral supply should be avoided whenever possible [23]. 

The guidelines recommend EEN in septic patients after achieving hemodynamic stability [1,2,3,4,5]. The MNT should be progressed slowly to achieve more than 80% of the nutritional target within the first week [3]. If EN is contraindicated, PN should be used. An SPN shall be administered according to the ESPEN after day 3 [2]. Contrasting the ESPEN guideline, the A.S.P.E.N. and Surviving Sepsis Campaign (SSC) do not recommend SPN or PN in the acute phase of a severe sepsis due to the limited substrate utilization [3,5]. Because septic patients in the hypermetabolic phase may have an increased need for vitamins and trace elements, these should be supplemented in physiological dosages after a proven deficit or of PN is needed [1]. Immonutrition, as well as micronutrients—such as selenium, glutamine, arginine, or carnitine—should not be administered [3,5].

Attention should be paid to the macronutrient balance in the first week of sepsis, because EN alone is often hypocaloric due to limited GI and metabolic tolerance. Whether this is advantageous or not remains controversial. The GI tolerance may be improved through prokinetics and postpyloric nutrition tubes, according to the SSC guideline [5].

The guidelines furthermore state, that patients with septic shock can receive EEN after stable hemodynamics are ensured (mean arterial pressure ≥60 mmHg and stable/falling lactate/need for vasopressors) but should progress slowly and be adapted to the patient’s tolerance [2,3]. In patients with uncontrolled shock, no EN should be given [2,3].

**Pragmatic recommendations:**

- **In hemodynamically stable patients, EN is preferable**
- **Careful monitoring of clinical and laboratory parameters is necessary**

### 10.5. Critically Ill Patients after Trauma and Burn Injury

Due to insufficient data in these patient groups, they should be treated according to the general recommendations for ICU patients [1]. The A.S.P.E.N. recommends EN should be preferred to PN [3].

Patients with burn injury and exudative losses through wound surfaces have an increased need for vitamins and trace elements. A highly variable EE leads to inaccuracy of estimation formulas regarding macronutrient needs. Therefore, indirect calorimetry shall be preferred [1,3]. The ESPEN recommends—contrasting the DGEM—enteral glutamine to be supplemented (0.3–0.5 g/kg/d) for 10–15 days [2] and the A.S.P.E.N. a higher dosage of protein (1.5–2 g/kg/d) [3]. According to DGEM, protein losses via drains and dressings should be compensated [1]. 

For trauma patients, the ESPEN recommends glutamine (0.2–0.3 g/kg/d) for the first 5 days (10–15 days if wound healing is complicated) [2]. Immunomodulating solutions with fish oil and arginine may be considered for patients after severe trauma according to the A.S.P.E.N. [3]. The DGEM advises against the use of these substrates in the sense of pharmacotherapy [1].

**Pragmatic recommendations:**

- **EN should be preferred**
- **Use indirect calorimetry as energy needs may be highly variable**
- **Consider supplementing more protein and micronutrients**

### 10.6. Critically Ill Patients with Central Nervous Diseases

The guidelines state that patients with head trauma, ischemic or hemorrhagic stroke and spinal trauma should receive EEN [2,3,4].

Patients with **stroke** are especially vulnerable for malnutrition, dehydration, and aspiration pneumonia due to impaired consciousness, swallowing problems as well as cognitive and perceptive deficits [119]. Therefore, all stroke-patients should be screened for swallowing problems and malnutrition and treated accordingly [120]. ONS may support the individualized therapy [119,120]. In patients, where impaired consciousness or dysphagia prohibit oral nutrition, an EN should be started within 72 h via nasogastric tube [119]. 

Young patients with **head trauma** are usually not malnourished at ICU admission but have an increased nutritional risk due to long ICU stays, variable EE (up to 200%) and frequently experienced a profound muscle catabolism. A higher protein supplementation 1.5–2.5 g/kg/d may therefore be considered [2,3]. The A.S.P.E.N. suggests the administration of formulas including arginine and omega-3-fatty-acids [3]. 

Patients with a diagnosed **swallowing disorder**, which make up to 60% of the general ICU population [121], should receive a logopedic therapy and an optimization of food texture to optimize oral food intake as stated in the ESPEN guideline “clinical nutrition in neurology” [119]. Special attention should be paid to oral and pharyngeal residue of food, sufficient food and fluid intake and aspiration [119,120]. If no safe and sufficient nutrition can be ensured, EN via nasogastric route shall be added to the oral nutrition [2,119,120]. If there is additional risk of aspiration, EN boles should be avoided, a postpyloric tube should be placed, the head should be elevated by 30°, and prokinetics should be considered [2,3,120]. PN is another option for these patients, especially in cases with preexisting malnutrition or if an EN is not sufficient to ensure adequate nutrition and hydration [2,120].

**Pragmatic recommendations:**

- **Increased risk of malnutrition during prolonged ICU stay, frequent re-assessment necessary**
- **EN is preferred, but often difficult due to paralytic ileus and increased risk of aspiration**

### 10.7. Critically Ill Patients with Cardiac Diseases

Malnutrition, cachexia, and sarcopenia are common comorbidities in patients with cardiac failure [17,122,123]. In contrast to often quickly recovering patients after elective “simple“ cardiac surgery, patients with preexisting malnutrition, complex heart surgery and elevated risk for prolonged ICU stays should receive regular screenings for nutritional risk [123,124]. At the same time, these patients are vulnerable for fluctuation of the volume status, which is why the use energy-dense formulae seems reasonable. 

For patients with mechanical assist devices (extracorporeal membrane oxygenation (ECMO)/ extracorporeal life support (ECLS), or ventricular assist devices (VAD)), the same recommendations apply as for other critically ill patients. According to the guidelines, in patients with stable hemodynamics and intact GI system, EN should be preferred [1,2,4]. Regarding monitoring, special attention should be paid to GI bleeding during therapeutic anticoagulation. In patients on ECMO/ECLS, diagnostic measures and therefore interruptions of EN are frequent. Although evidence remains sparse, SPN seems safe and should be infused via central venous catheter (as opposed to directly into the ECMO circuit [1]. Indirect calorimetry is not reliable in this patient group due to the extracorporeal CO_2_-elimination, therefore weight-based formulas should be used to estimate energy targets [1].

**Pragmatic recommendations:**

- **Heterogeneous patient group, focus MNT on patients with increased risk of malnutrition**
- **EN is preferred**
- **Special attention should be paid to hemodynamic stability and fluid status**

### 10.8. Critically Ill Patients with Respiratory Diseases

In patients with respiratory failure, in theory, formulae with increased fat content and reduced carbohydrates may be useful to influence the respiratory quotient and decrease CO_2_-production. The A.S.P.E.N. advises against these formulae in patients with acute respiratory failure due to the lack of evidence, but recommends avoiding hyperalimentation, because lipogenesis increases CO_2_-production [3]. 

According to the guidelines, EN should not be administered to patients with life-threatening hypoxia, hypercapnia, and acidosis as sign of respiratory decompensation. In patients with stable and compensated respiratory failure, EN can be commenced [2,4]. A restriction of fluids seems reasonable to avoid aggravating overhydration and edema. Therefore, energy-dense formulae (1.5–2 kcal/mL) are recommended [3]. Hypophosphatemia is a common (and commonly unrecognized) problem and may lead to weakness of respiratory muscles and weaning-failure. Therefore, phosphate should be monitored closely and replaced as necessary [3]. A functional GI-System should be used, so EEN should be performed in patients managed in prone position [2]. The hypothesis that the abdominal compression leads to problems with transport and resorption has not been proven [1,2,4]. One possibility to increase tolerance and avoid aspiration of EN is to bring the entire bed in a 30° head elevation. Measurements of intraabdominal pressure may aid in the early detection of an abdominal/GI problem (not only) in patients in prone position. 

#### Critically Ill COVID-19 Patient

Most COVID-19 patients admitted to the ICU are at high risk of or have preexisting malnutrition [125]. According to Ochoa et al., COVID-19 patients present with three different phenotypes of nutrition risk: (1) the frail older patient, (2) the patient with severe ongoing chronic illness, and (3) the patient with severe and morbid obesity [126]. The measurement of the upper waist circumference of COVID-19 patients has recently shown that with every centimeter increase there is a 1.13-fold higher probability of intensive treatment and a 1.25-fold higher probability of mechanical ventilation [127].

Regarding MNT in this patient cohort, no RCTs exist so far. Instead, the ESPEN and A.S.P.E.N. have published expert statements as an adaptation of their existing guidelines [128,129].

Before admission to the ICU, anorexia secondary to infection, dyspnea, dysosmia, dysgeusia, and impaired meal preparation during quarantine may have reduced food intake. Therefore, upon ICU admission, nutritional assessment is mandatory. An individualized approach including indirect calorimetry is recommended, because persistent hypermetabolism was observed in these patients [130,131]. 

While EN may be performed even in prone position, Martindale et al. recommend a lower threshold for switching to PN in cases of intolerance, high risk of aspiration, or escalating vasopressor support [129]. In case of ARDS, Thibault et al. have recommended the use of EN enriched with omega-3 fatty acids, and for PN fish oil-enriched intravenous fat emulsions [125]. 

In a multicenter, double-blind, RCT conducted at two sites in Sao Paulo, Brazil, 240 hospitalized patients with COVID-19 being moderately to severely ill were randomized to receive a single oral dose of 200,000 IU of vitamin D3 or placebo [132]. LOS defined as the primary endpoint was not significantly different between the vitamin D3 and placebo groups (log-rank *p* = 0.59; unadjusted hazard ratio for hospital discharge, 1.07 [95% CI, 0.82 to 1.39]; *p* = 0.62). Furthermore, there were no significant differences for in-hospital mortality, admission to the ICU (*p* = 0.30), or need for mechanical ventilation (*p* = 0.09). Mean serum levels of 25-hydroxyvitamin D significantly increased after a single dose of vitamin D3 vs. placebo (44.4 ng/mL vs. 19.8 ng/mL; difference, 24.1 ng/mL [95% CI, 19.5 to 28.7]; *p* < 0.001).

**Pragmatic recommendations:**

- **EN is preferred in patients with respiratory problems including COVID-19 and ARDS patients, as well as patients in prone position**
- **Do not use EN in patients with respiratory decompensation**

### 10.9. Critically Ill Patients with Abdominal Diseases

Critically ill patients requiring abdominal surgery often present anatomical and functional characteristics that require a critical evaluation and adaptation of MNT. Compromised functions are GI-motility, digestion, and absorption of nutrients, which often leads to a reduced tolerance of EN. On the other hand, EN also nurtures the gut mucosa, increases intestinal perfusion and peristalsis [3,17].

#### 10.9.1. Patients after GI-Surgery

If the GI system is functional, it shall be used in patients after **GI surgery** as recommended uniformly by all guidelines [1,2,3,4]. Therefore, in most abdominal-surgery patients an EEN within 24–48 h is recommended [1,12]. Even if the nutrition of these patients remains a controversially debated topic, it is not necessary to withhold EN as per standard or administer clear liquids only [3]. The MNT should be discussed in the interdisciplinary teams to optimize the nutrition for each individual patient [12].

In patients after **upper GI-surgery,** an intraoperatively placed nasojejunal or percutaneous postpyloric tube can allow for EN of the distal GI parts without risking injury of a fresh anastomosis during feeding tube placement and without risk of regurgitation and aspiration. In general, fresh anastomoses *per se* are not a contraindication for EN, whereas the individual therapy needs to be decided in the interdisciplinary team [2].

Abdominal surgery patients with **complicative courses** often accumulate great energy debts. Therefore, the ESPEN recommends to consider an early SPN [2]. In patients with leaky anastomoses, with internal or external fistulas, an access to the distal part of the gut shall be used for EN. Enteroclysis shall be considered and re-evaluated on a regular basis, to increase resorption of nutrients and prevent mucosal atrophy as well as bacterial overgrowth with the risk of bacterial translocation and bacteriemia [2]. If EN is insufficient, the patient needs PN [2].

Patients with **open abdomen** should receive an EEN (24–48 h postinjury) in the absence of bowel injury [3,4]. Protein losses via drains and dressings should be compensated in the form of enteral protein supplements or parenteral albumin (15–30 g protein/ liter exudate) [1,3]. In addition to the above-mentioned guideline recommendations, an algorithm was proposed by Friese et al. in 2012 [133] and Moore and Burlew in 2016 [134]. 

#### 10.9.2. Patients with Liver Failure

According to the ESPEN guideline on clinical nutrition in liver disease [135] and the A.S.P.E.N. [3], patients with liver failure have an increased risk for malnutrition and may develop severe disturbances regarding carbohydrate, protein and fat metabolism with impeded hepatic gluconeogenesis and lactate clearance, as well as catabolism [3,135]. Therefore, all patients with liver disease should be screened for malnutrition and treated accordingly [135]. EE may be highly variable in these patients; therefore, indirect calorimetry should be used [3,135]. If the latter is not possible, the patient’s dry weight shall be used to estimate energy and protein targets and 1.3× resting EE should be supplied [135]. Patients with acute liver failure should receive oral nutrition as long as possible, or in case of hepatic encephalopathy, EEN [135]. A generalized protein restriction is not recommended prevent muscle degradation, which contributes to the development of a hepatic encephalopathy [3]. In patients with encephalopathy and high ammonia, the protein supplementation can be delayed for 24–48 h [135]. A normal EN formula shall be used, because of insufficient evidence for the use of branched chained amino acids [3,135]. 

#### 10.9.3. Patients with Acute Pancreatitis

In patients with acute pancreatitis, there is a great range of severity. Therefore, the MNT should be re-evaluated frequently. In mild cases, patients can receive oral nutrition ad libitum. In medium or severe cases, EEN should be commenced with a low infusion rate via gastral or jejunal path. If the patient undergoes surgery for necrosectomy, placement of needle catheter jejunostomy should be considered [136]. Before PN is considered one week after onset of pancreatitis, measures to increase GI-tolerance can be performed, according to A.S.P.E.N. [3].

**Pragmatic recommendations:**

- **Patients with abdominal diseases have a high risk for malnutrition**
- **MNT in patients with abdominal diseases often requires an interdisciplinary team**
- **EN is the preferred route in patients with liver failure, acute pancreatitis, after GI surgery and may be used even in patients with enteric fistulas and/or open abdomen.**

### 10.10. Critically Ill Patients with Renal Diseases

Patients with renal failure represent a heterogeneous group with different needs regarding macro- and micronutrients. EN should be preferred in this patient group. If an EEN is not possible, SPN or TPN should be started early according to the German guideline for Patients with Kidney Disease [137]. 

A patient with acute kidney failure has the same needs for energy and protein and should be treated according to the standard of other critically ill patients—compared to patients with chronic kidney failure, who have increased energy demands as stated in the guidelines [1,3,137]. Even when deranged values for potassium and phosphate are rare in patients with acute-on-chronic kidney failure, a careful electrolyte monitoring is important. In case of electrolyte derangements and no indication for RRT, special renal formulae can be used [3]. These formulae contain less fluid and protein, are high in calories and have lower potassium and phosphate content and can contain additional substances such as carnitine [137].

A RRT increases losses of energy, water soluble molecules—such as amino acids, electrolytes, trace elements, and vitamins—and induces systemic inflammation and protein catabolism. On the other hand, substances sch as lactate and citrate are added excessively with the dialysis or hemofiltration solution. This needs to be minded for calorie calculations. The guidelines vary in their recommendations regarding energy supplementation for ICU patients with kidney failure during RRT. The DGEM guideline recommends macronutrient calculation as per the standard in other ICU patients, which should be ramped up slowly while considering macronutrient losses. The A.S.P.E.N guideline suggests administration of high protein dosages of 2.5 g/kg/d, to achieve nitrogen balance [1,3,137]. To cope with the increased need of vitamins and trace elements, an increased supply is recommended, with special regard to vitamin C, folate and thiamine [1,137]. 

**Pragmatic recommendations:**

- **Use the same principles as in other ICU patients regarding macronutrient requirements, timing, and route**
- **In patients on RRT, an increased supply of amino acids, electrolytes vitamin, and trace elements should be considered**
- **Electrolytes need to be monitored closely**

## 11. Summary and Conclusions

Despite ongoing research activities, the level of evidence remains often low due to a lack of data from large RCTs. 

Evidence based international guidelines are available. Nevertheless, the implementation of the different recommendations into clinical routine remains often insufficient. 

It should be kept in mind that every ICU-patient is at risk for malnutrition, regular screenings for malnutrition should be performed, close monitoring and frequent adaptation of nutrition is necessary. An early enteral nutrition with a standard formula is preferred in almost all critically ill patients. The addition of parenteral nutrition and micronutrients should be considered individually. Feeding protocols which are tailored to the treating units will improve the nutritional performance. 

Due to the heterogeneity of the patients, MNT should be carefully adapted to the individual patient with special focus on phase of critical illness, metabolic tolerance, leading symptoms, and comorbidities. Nutrition in the ICU is a complex therapy requiring an interdisciplinary approach and frequent reevaluation. This article can only be regarded as an introduction and summary for the complex topic of nutrition in the critically ill patient and may be of help for the clinical routine. 

Appreciating recent advances, the long-lasting open questions will remain:

- The optimal nutritional assessment and monitoring
- The impact of personalized and disease-specific MNT on patient-centered clinical outcomes
- The impact of high vs. low protein and energy intake according to the phase of illness

## Figures and Tables

**Table 1:** Current guideline recommendation regarding micronutrients, vitamins, and antioxidants.

Substrate	DGEM	ESPEN	A.S.P.E.N.
Micro-nutrients	A patient should receive vitamins and trace elements, if EN cannot meet daily needs, and if supplemental PN is required to ensure the desired calorie and protein intake according to the disease phase and individual metabolic tolerance.	To enable substrate metabolism, micronutrients (i.e., trace elements and vitamins) should be provided daily with PN.	N/A
Vitamin D	Patients may receive a pharmacotherapy with vitamin D, when they have a severe vitamin D deficiency (25 [OH] D ≤ 30 nmol/L corresponding to 12 ng/mL).	In critically ill patients with measured low plasma levels (25-hydroxy-vitamin D < 12.5 ng/mL, or 50 nmol/L) a high dose of vitamin D3 (500,000 UI) as a single dose can be administered within a week after admission.	N/A
Antioxidants/ Selenium	Patients shall not receive a pharmacotherapy with selenium. Patients should not receive routinely a pharmacotherapy with zinc, alpha-tocopherol, vitamins A and C, or with a combination of those.	Antioxidants as high dose monotherapy should not be administered without proven deficiency.	We suggest that a combination of antioxidant vitamins and trace minerals in doses reported to be safe in critically ill patients be provided to those patients who require specialized nutrition therapy. We cannot make a recommendation regarding selenium, zinc, and antioxidant supplementation in sepsis at this time due to conflicting studies.

DGEM, German Society for Nutritional Medicine; ESPEN, European Society of Enteral and Parenteral Nutrition; A.S.P.E.N, American Society of Enteral and Parenteral Nutrition.

## Data Availability

Not applicable.

## Abbreviations

A.S.P.E.N	American Society of Enteral and Parenteral Nutrition
BIA	Bioelectrical Impedance Analysis
BMI	Body Mass Index
CT	Computed Tomography
DGEM	Deutsche Gesellschaft für Ernährungsmedizin (German Society for Nutritional Medicine)
EN	Enteral Nutrition
EN+PN	Combined Enteral and Parenteral Nutrition
ESICM	European Society of Intensive Care Medicine
ESPEN	European Society of Enteral and Parenteral Nutrition
GI	Gastrointestinal
GLIM	Global Leadership Initiative on Malnutrition
ICU	Intensive Care Unit
LOS	Length of Stay
MNT	Medical Nutrition Therapy
MUST	Malnutrition Universal Screening Tool
NRS 2002	Nutrition Risk Score 2002
NUTRIC	Nutrition Risk in the Critically Ill Score
ONS	Oral Nutritional Supplements
PN	Parenteral Nutrition
RCT	Randomized Controlled Trial
RRT	Renal Replacement Therapy
SPN	Supplementary Parenteral Nutrition
SGA	Subjective Global Assessment

## Appendix A

**Table A1:** Weight based calculation of macronutrient requirements, *, recommendation for acute phase, use actual body weight for calculation.

Guideline	Energy in kcal/kg/d	Protein in g/kg/d
DGEM [1]	24 *	1.0–1.2 *
ESPEN [2]	20–25	1.3
ASPEN [3]	25–30	1.2–2
